# Innovative application of an implantable venous access system in the portal vein: technique, results and complications in three dogs

**DOI:** 10.1186/s12917-019-1986-6

**Published:** 2019-07-11

**Authors:** Kiona S. de Nies, Hedwig S. Kruitwagen, Giora van Straten, Leonie W. L. van Bruggen, Joris H. Robben, Baukje A. Schotanus, Ies Akkerdaas, Anne Kummeling

**Affiliations:** Faculty of Veterinary Medicine, Departement of Clinical Sciences of Companion Animals, Utrecht University, Yalelaan 108, 3584 CM Utrecht, The Netherlands

**Keywords:** Dogs, Implant, Portal vein, Thrombus, Vascular access port

## Abstract

**Background:**

Vascular access port (VAP) systems are widely used in human medicine to provide long-term venous access. However, in veterinary medicine the use of VAP systems is not common practice and publications on their potential applications have been limited. A VAP system was used as part of an experimental study on liver regeneration and implanted in the canine portal vein to create direct access to the portal venous circulation of the liver. The aim of the present study is to describe the surgical technique, its use, and the complications of a VAP system in three research dogs.

**Results:**

The VAP system was successfully used for the intraoperative measurement of portal blood pressure, the administration of cell suspensions, and the collection of portal venous blood samples. Long-term complications consisted of dislocation of the VAP system in one dog (2 months after implantation) and thrombus formation at the catheter tip in two dogs (3 months after implantation). Both complications prevented further use of the VAP but had no adverse clinical implications.

**Conclusions:**

This pilot study suggests that the VAP system is an effective and safe technique to obtain long term access to the portal venous system in dogs. However, complications with port detachment and thrombosis may limit long term use of VAPs in the portal system of dogs.

## Background

This study describes a novel use in dogs of a vascular access port (VAP) surgically implanted into the portal vein to create direct access to the portal venous circulation of the liver. Vascular access port systems are widely used in human medicine to provide long term central venous access in patients who require comprehensive intravenous fluid therapy, multiple administrations of medication or repeated blood sampling. In people, they are commonly implanted in the superior vena cava via a subclavian, cephalic or jugular vein [1–3]. In veterinary medicine the use of VAP systems is not common practice and literature on applications has been limited. Due to the reduced risk of infection, the limited chance for dislodgement and the ability to provide minimally invasive vascular access for repeated and long-term use, implantable VAP systems were previously suggested for both animal and human use [4–6]. VAP systems in the dog and cat can be valuable tools in experimental research, but also have clinical potential in veterinary medicine [4, 7–10]. VAP systems are described to be useful for chronic intravenous drug therapy, long-term use of chemotherapeutics and blood sampling [4, 11, 12]. Also, VAP systems were used in radiotherapy protocols requiring frequent anaesthesia to provide long-term venous access [5–7].

VAP implantation in the portal vein was advocated for regional chemotherapy of liver metastasis [2]. Permanent portal vein catheterization using a VAP was described in a human case series of repeated hepatocyte transplantations via the portal vein [8]. Use of VAP systems in canine portal veins has not been reported yet, as far as the authors know.

A VAP system in dogs has been evaluated as part of a research project in which liver cells were transplanted into the liver via the portal vein ‘unpublished observations by H.S. Kruitwagen et al.’. The VAP system was intended to provide long-term, low-invasive portal venous access for the administration of cell suspensions and the collection of portal venous blood samples. The possibilities for direct measurement of portal venous blood pressure were also explored. The aim of this preliminary report is to describe the surgical technique, results, and complications of a VAP system in three dogs.

## Methods

### Study design

All procedures were approved by Utrecht University’s Ethical Committee, as required under Dutch legislation (DEC number 2014.III.12.112) and performed at the Utrecht University Clinic for Companion Animals. Three research dogs were instrumented with the VAP system and the system was evaluated for a subsequent period of 5 months. After surgical implantation, the VAP system was used for intravenous infusion of liver cells intraoperatively and once daily for 2 days after surgery. The VAP system was also used for direct intraoperative measurement of portal venous pressure.

### Dogs

Venous access port systems were implanted in three Beagle-Bedlington terrier crossbreeds with copper toxicosis due to COMMD1 deficiency [9]. Dog no. 1 was an intact male (5 years, 15.0 kg), dog no. 2 an intact male (8 months, 15.5 kg), and dog no. 3 an intact female (8 months, 10.6 kg).

### Anaesthesia and monitoring

Pre-operatively, all dogs received cefazolin 20 mg/kg IV (Kefzol, Eurocept International B.V., Ankeveen, The Netherlands). The dogs were premedicated with glycopyrrolate 0.01 mg/kg IM (Robinul 0.2 mg/mL injection, Riemser Pharma GmbH, Greifswald, Deutschland) and methadone 0.5 mg/kg IV (Comfortan 10 mg/mL, Dechra, Bladel, The Netherlands). Propofol 1–4 mg/kg dosed to effect IV (Propofol 1%, MC Fresenius Kabi, Zeist, The Netherlands) was used for induction. After intubation, general anaesthesia was maintained with isoflurane dosed to effect in a mixture of oxygen and air 1:1 and fentanyl continuous rate infusion 10–20 μg/kg/h CRI (Fentanyl 0.05 mg/mL, Bipharma, Almere, The Netherlands).

Postoperatively, the dogs were admitted to the intensive care unit for analgesia and monitoring. A clinical examination including abdominal circumference and central venous pressure measurements were performed every 4 hrs for 3 days. Mean arterial pressure was monitored via an intra-arterial line for at least 24 h postoperatively. Venous blood analysis was performed pre-operatively, and 1 day, 2 days, 3 days, 1 week, 1 month, and 3 months post-operatively. Blood was sampled through the jugular catheter for the first three postoperative days and at later time points by venipuncture and was analysed for the following parameters: haematocrit, platelet count, activated partial thromboplastin time (aPTT), prothrombin time (PT), fibrinogen, total protein and albumin. Dogs received postoperative pain medication, initially consisting of fentanyl 3–5 μg/kg/h CRI (Fentanyl, Bipharma, Almere, The Netherlands) and ketamine 3–5 μg/kg/min CRI (Narketan, Vetoquinol, ‘s-Hertogenbosch, The Netherlands) to effect. After two to 3 days analgesia was continued for 3–5 days with tramadol 3–5 mg/kg four times daily per os (Tramadol HCl, Centrafarm, Etten-Leur, The Netherlands). As part of the liver cell transplantation protocol, dogs received cyclosporine 6.25 mg/kg two times daily per os (Sporimune, AST Farma, Oudewater, The Netherlands) from the day before surgery until 3 months post-operatively.

### Surgery

First, the implantation of the VAP system was practiced on two fresh cadavers of female mixed breed dogs (28 kg), euthanized for non-related medical experiments (surplus material, Utrecht University 3R-policy). The surgical protocol was then applied to the three experimental dogs. All surgeries were performed by the same surgeons. A midline celiotomy was performed in dorsal recumbency and the falciform ligament was removed after cranial ligation. A left lateral hepatic lobectomy was performed as part of the liver cell transplantation study using double ligation of the large vessels near the hilus of the lobe with polydioxanone (PDS 0, Johnson & Johnson International, Amersfoort, The Netherlands) and additional electrocoagulation to seal the parenchyma peripheral to the ligated vessels.

**Fig. 1:**
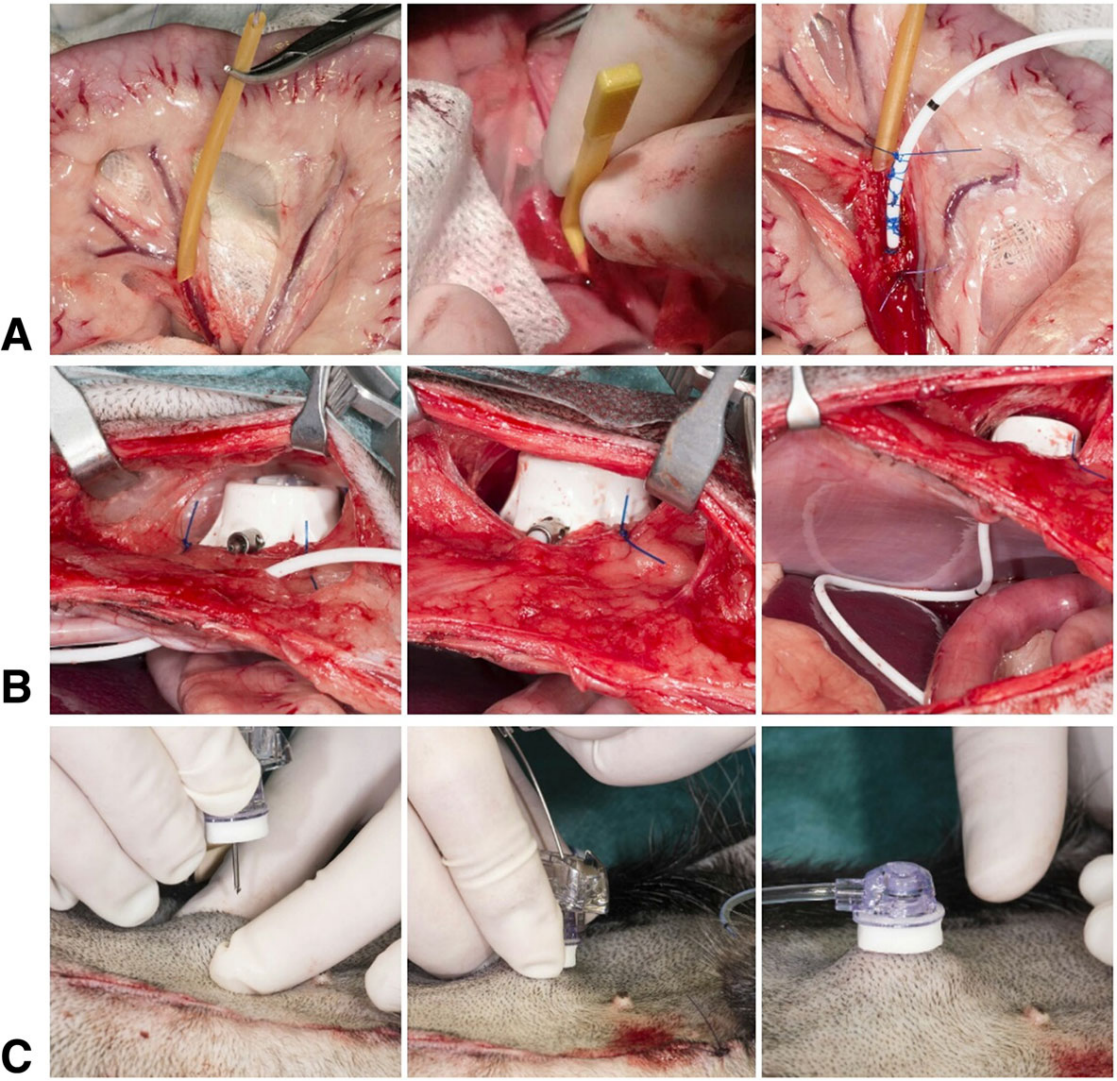
Surgical implantation of a vascular access port (VAP) into the portal venous circulation of the liver: **a** Vascular access catheter implantation: selection and isolation with a Rummel tourniquet of a jejunal vein in the omentum (A, left); usage of a vein hook to obtain access and introduce the catheter into the jejunal vein (A, middle); fixation of the catheter using a Chinese finger trap suture (A, right). **b** Subcutaneous implantation of the portal. **c** Percutaneous placement of the gripper needle into the subcutaneous portal

The VAP system used was a PORT-A-CATH II POWER system (reference number: 21–4477-24, Smiths Medical Nederland B.V., Rosmalen, The Netherlands) that consisted of a polyurethane catheter with an outer diameter of 1.9 mm (5.8 Fr), a titanium connector and a single lumen portal made of polysulfone and titanium. The priming volume was provided by the manufacturer and manually confirmed pre-operatively. The volume amount of the catheter + portal = 0.7 + 0.3 ml = 1 ml. This is the total amount needed to fill a non-adjusted device. The VAP system was prepared and flushed with heparinized saline (50 IU/mL). A distal branch of the extrahepatic portal circulation (jejunal or splenic vein) was selected based on accessibility and size (Fig. 1). Two polyglecaprone 25 (Monocryl 4–0, Johnson & Johnson International, Amersfoort, The Netherlands) stay-sutures and a Rummel tourniquet (Fig. 1a, left) were placed around the vessel to fixate the vessel and to prevent bleeding. A stab incision was made between both sutures and elongated to approximately 5 mm over the length of the vein. With the aid of a vein hook (Fig. 1a, middle) the rounded tip of the VAP catheter was inserted into the vein and carefully fed towards the main stem of the extrahepatic portal vein, just one to two centimetres proximal to the hepatic entrance. The position was checked by palpation and the VAP catheter was fixed at the insertion site using a Chinese finger trap suture technique with polypropylene (Prolene 4–0, Johnson & Johnson, Amersfoort, The Netherlands) (Fig. 1a, right). The tourniquet was removed and the preplaced stay-sutures, one distal and one proximal to the catheter insertion, were tied. The VAP system was repeatedly flushed with heparinized saline (50 IU/mL).

The port of the VAP system was placed in a subcutaneous pocket lateral to the caudal part of the midline incision. A stab incision was made in the abdominal wall and the extravenous part of the catheter was inserted through the incision and connected to the portal outside the abdomen. The portal was fixated to the muscular fascia with three polypropylene (Prolene 2–0, Johnson & Johnson, Amersfoort, The Netherlands) interrupted sutures (Fig. 1b). The VAP system was flushed again to confirm patency and the subcutaneous pocket was closed with single interrupted polyglecaprone 25 sutures. A 19G gripper needle (reference number: 21–3280-24, Smiths Medical Nederland B.V., Rosmalen, The Netherlands) was placed percutaneously into the port to obtain venous access for the liver cell infusions and to enable portal venous pressure measurements (Fig. 1c). The system was flushed with saline to confirm patency. The abdomen was closed routinely.

### Portal pressure measurements

We explored the possibility to measure the pressure in the portal vein directly and non-invasively via the VAP system. For this purpose, an extension tubing was connected to the gripper needle in the subcutaneous portal during surgery in two dogs. Venous pressure was measured by a water column method in centimetres H_2_O and electronically by a Merit Medical single transducer set (Gabarith® PMSET 1DT-XX 1rose) connected to a Datex-Ohmeda S5 Anaesthesia Monitor in millimetres Hg. In both cases, the level of the right atrium was used as zero reference.

### Maintenance and patency checks of VAP

Patency of the VAP system was checked by flushing portal and catheter with saline once daily on the first 2 days after surgery before and after infusing cells through the VAP into the portal bloodstream. Images of the portal vein and the tip of the catheter were obtained by B-mode imaging and colour Doppler was applied to check for hepatopetal flow during infusion. The gripper needle was removed 5 days after surgery.

Subsequently, patency was checked by flushing the VAP system once a month. The skin overlying the portal was clipped, locally anesthetized with a combined lidocaine and prilocaine ointment EMLA Cream 5% (AstraZeneca, Den Haag, The Netherlands) and routinely disinfected. A 19G gripper needle (reference number: 21–3468-24, Smiths Medical Nederland B.V) was inserted percutaneously into the subcutaneous portal. Using a syringe, 5 ml of blood was withdrawn and the catheter was subsequently flushed with 10 ml of saline. Patency of the portal vein and the position of the intravenous catheter were also checked with abdominal ultrasonography. After each use of the VAP, a heparin lock was placed by infusion of 5 ml of heparinized saline (100 IU/ml).

## Results

### VAP system implantation

In dog no. 1, the VAP catheter was successfully inserted via a mesenteric jejunal vein. In dog no. 2 and 3 mesenteric jejunal vein insertion was unsuccessful, as the jejunal veins proved to be too small in diameter. Therefore, in dogs no. 2 and 3 the VAP insertion was performed in a splenic vein without any problems.

### Portal pressure measurements

In dog no. 2 and 3, portal pressure could be measured intraoperatively via the VAP system both via a water column method and electronically. Portal pressure was 13 cmH_2_O and 10 mmHg, respectively for dog no. 2, and 13 cmH_2_O and 14 mmHg for dog no. 3.

### Post-operative complications

Dogs no. 1 and 2 developed a haemoabdomen within 24 h after surgery, confirmed with ultrasound guided abdominocenthesis. Explorative celiotomy showed diffuse bleeding from the hepatic lobectomy site, which was controlled with additional electrocoagulation and ligation. Both dogs recovered completely with additional supportive care including packed red cell and plasma transfusions. Results of blood analysis are presented in Table 1. All three dogs experienced a decrease in haematocrit, plasma total protein and albumin levels consistent with blood loss.

**Table 1:** Blood parameters in three dogs as measured pre-operatively (day of surgery) and up to three months post-operatively (PO)

	Dog	Day of surgery	1 day PO	2 days PO	3 days PO	7 days PO	1 month PO	3 months PO
Hematocrit (RI: 0.42–0.61 L/L)	1	0.45	0.39	0.20	0.35	0.37	NA	0.47
	2	0.43	0.23	0.35	0.36	0.41	0.38	0.43
	3	0.50	0.29	0.32	0.36	0.45	0.48	0.45
Platelets (RI: 144–603 10^9/L)	1	287	212	57	NA	205	468	372
	2	556	70	96	107	164	299	178
	3	569	116	124	148	136	244	NA
aPTT (RI: 13.2–18.2 s.)	1	17.2	15.7	19.8	27.9	18.7	14.1	15.9
	2	15.9	NA	28.3	NA	17.1	15.4	15.1
	3	13.2	16.4	18.4	NA	13.3	13.2	14.3
PT (RI: 7.2–9.9 s.)	1	7.4	9.6	9.8	9.7	9.7	7.9	7.6
	2	7.7	NA	16.6	NA	9.0	8.3	7.9
	3	7.2	8.7	9.2	NA	7.7	7.8	7.4
Fibrinogen (RI: 1.0–2.7 g/L)	1	2.9	3.2	3.3	4.7	4.4	4.3	2.4
	2	4.8	NA	2.3	NA	1.7	2.1	2.0
	3	2.2	3.5	4.4	NA	2.2	1.8	2.5
Total Protein (RI: 55–72 g/L)	1	65	42	35	40	55	NA	72
	2	55	32	39	49	NA	53	55
	3	61	39	49	50	65	61	62
Albumin (RI: 26–37 g/L)	1	31	19	16	16	21	NA	34
	2	29	15	20	25	NA	28	28
	3	30	20	23	23	31	29	29

Abbreviations: *RI* reference interval, *aPTT* activated partial thromboplastin time, *PT* prothrombin time, *NA* not available

### Postoperative use of the VAP system

**Fig. 2:**
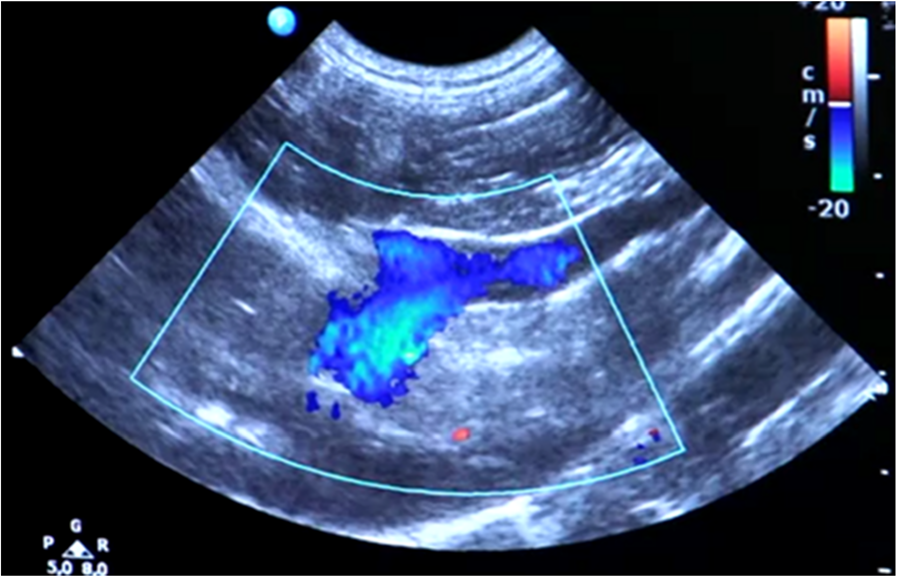
Doppler-enhanced ultrasonography visualizes hepatopetal flow in the portal vein at the level of the porta hepatis during infusion of liver cell suspensions via the vascular access port

**Fig. 3:**
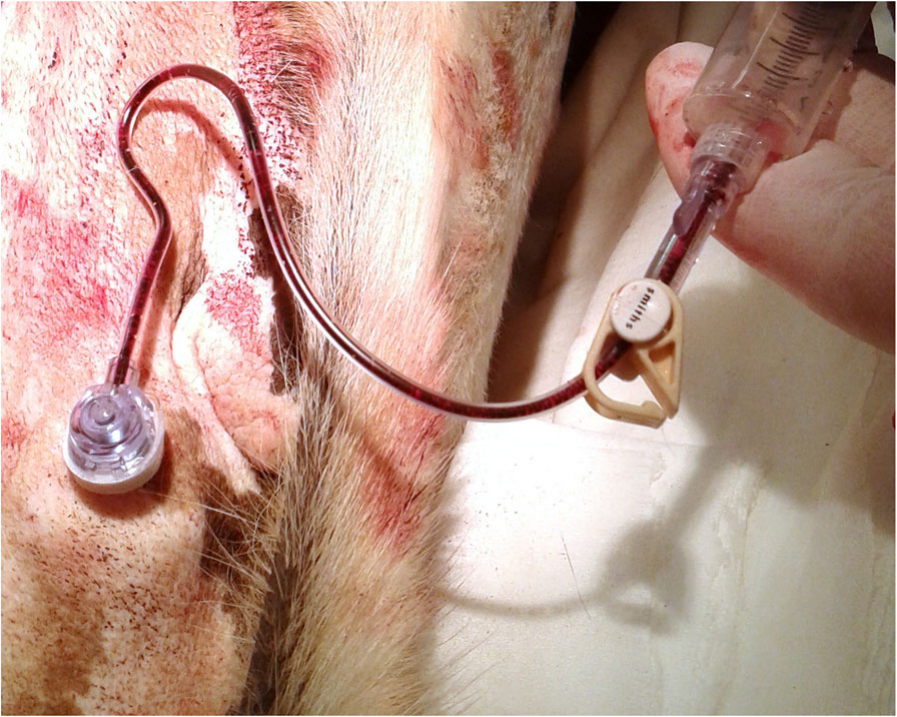
Sampling of portal blood using the gripper needle inserted percutaneously in the subcutaneous portal in a dog

In all dogs, portal vein injections (Fig. 2.) and blood sampling (Fig. 3.) via the VAP system using the gripper needle did not require sedation and did not result in any clinical adverse events. Withdrawal of portal blood via the VAP was possible up to 3 months after surgery in dog no. 2, and in dog no. 1 and no. 3 up to 1 month after surgery.

### VAP system complications

**Fig. 4:**
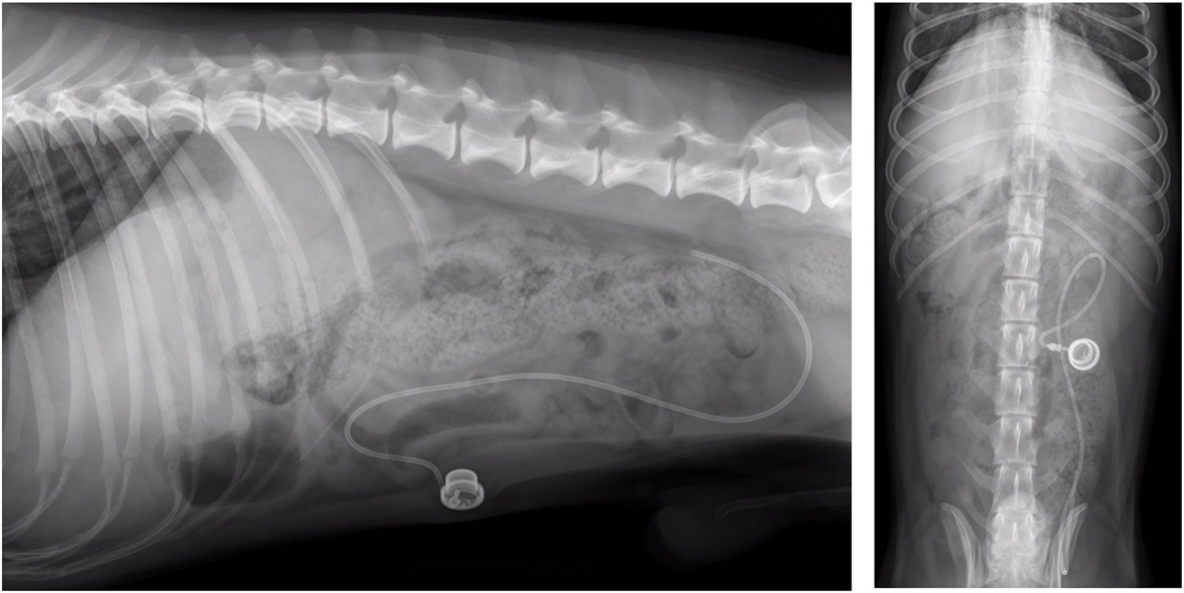
Vascular access port (VAP) system complication in dog no. 1. Abdominal radiographs show the venous catheter of the VAP system dislocated from the portal vein two months after surgical implantation. The tip of the catheter is located caudodorsally in the abdomen

**Fig. 5:**
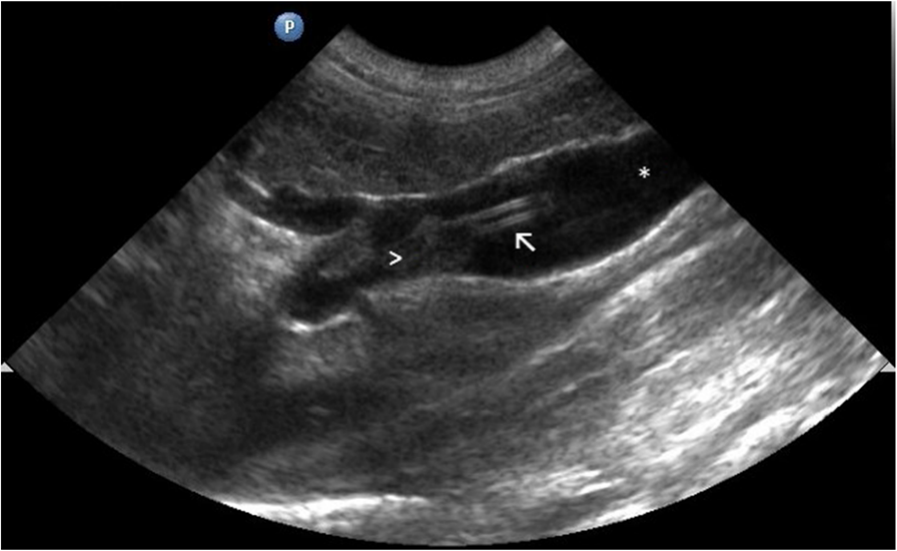
Vascular access port (VAP) system (PORT-A-CATH® II POWER system; Smiths Medical, Ashford, Kent, UK) complication in dog no. 2. B-mode image shows thrombus formation (arrow head) at the level of the catheter tip (arrow) four months after surgical implantation. The tip of the catheter is situated in the portal vein (asterisk)

In all dogs the VAP system became dysfunctional one to 4 months after placement. This did not result in any adverse clinical signs. In dog no. 1 routine ultrasonographic evaluation 2 months after placement showed the catheter tip to be dislocated from the proximal position in the portal vein. The exact location of the VAP catheter was determined with additional abdominal radiographs (Fig. 4a,b) and computed tomography (PhilipsSecura, single-slice helical CT scanner; Philips NV, Eindhoven, The Netherlands). The catheter was lying free within the abdominal cavity. In dogs no. 2 and 3 the catheter became dysfunctional four and 1 month after placement, respectively, because of an obstruction due to thrombus formation at the tip of the catheter, extending into the lumen of the portal vein (Fig. 5). After the loss of VAP system functionality, the VAP was removed in all dogs during celiotomy without complications at 107, 156 and 142 days after implantation for dogs no. 1, 2 and 3, respectively. After removal of the catheter, the vein was ligated distal to the insertion site.

## Discussion

This study describes the implantation and use of a commercially available VAP system in the portal venous system of three dogs. The VAP system was successfully used for administration of cell suspensions in the portal vein and blood sampling from the portal vein for a minimum period of 1 month after placement. It was demonstrated that also direct portal blood pressure measurements during surgery are possible with the use of the VAP system, and the results are within the reported physiological range for dogs [13, 14].

In contrast to percutaneous venous catheters, VAP systems have a subcutaneous portal access that offers better protection against external trauma, such as auto-mutilation, and contamination. Their potential durability enables continuous intravenous access for a longer time period, both for clinical and research purposes [11, 12, 15–18]. This study demonstrates their effectiveness in obtaining easy and long term access to parts of the cardiovascular system that are out of reach of percutaneous catheters. Therefore, VAP systems could be useful in a wide range of clinical and research applications in dogs such as prolonged or frequent intravenous administration of therapeutics, blood pressure measurements or blood sampling.

For access to the portal venous system both the use of a jejunal vein and a splenic vein as insertion site for the VAP catheter have been advocated [17, 18]. The use of a five to seven French diameter catheter has been suggested in dogs [18]. The 5.8 French diameter catheter that was used in this study still appeared too large for insertion in a jejunal vein in two medium sized dogs. However, a splenic vein offered a good alternative for catheter insertion when the jejunal veins were too small.

Postoperatively, haemoabdomen was a serious complication in dog no.1 and no.2. In both dogs no signs of a local bleeding were observed due to ineffective ligation of large hepatic vessels and both showed coagulopathy shortly after surgery (Table 1). Retrospective evaluation of the procedures noticed an extensive use of heparin in both dogs: heparinised sodium chloride 0.9% with a concentration of heparin of 50 IU/ml was used to flush the IV catheters (VAP system, peripheral and central venous catheters) very often. The exact amounts of administered heparin were not documented but overuse could have iatrogenically caused a hypocoagulable state. Therefore in dog no. 3 the heparin concentration in saline used to flush all catheters per-operatively was reduced from 50 to 10 IU/ml and flushing volumes were minimized. No postoperative coagulopathy or other complications were noticed in dog no. 3.

Infection of the VAP system is one of the most common complications in human patients [19], and in animals [5, 12, 15, 17, 18]. Monthly microbial cultures of specimens from the port revealed a rate of one infection per 200 catheter days [17]. In this study no microbial cultures were performed, but no clinical signs indicating infection (e.g. fever, leucocytosis) were encountered in our dogs. In future applications, microbial cultures could be useful to detect early (subclinical) infections.

Dislocation of the catheter or port has been described as a complication in both veterinary and human use [8, 11, 18, 19]. In dog no. 1 the catheter was dislocated from the prolene suture, which was still intact. It is unclear why the suture did not hold in this dog, but dislocation of the catheter may have been related to the suture technique, the thermoplastic nature of the polyurethane catheter or motility of the intestines [20]. The fact that the catheters positioned in the splenic vein did not dislocate supports the causative role of intestinal motility on the dislocation. For future VAP use in the portal system in dogs we would therefore recommend a splenic vein as first choice insertion site.

Although no adverse clinical signs developed by the thrombosis at the tip of two VAP catheters, deep venous thrombosis of the portal vein with complete intraluminal obstruction can lead to portal hypertension and hepatic embolization [21]. Conceivably, we noticed the complication early in its development before portal hypertension could occur. Few cases of VAP system-associated venous thrombosis have been reported in veterinary medicine [11, 18]. In an experimental study with a VAP system in the portal system no thrombosis of the portal vein was reported [17]. Differences between studies in the occurrence of thrombi may be explained by differences in species, the applied VAP system, its vascular location, the applied (chemo)therapeutics, time span and catheter maintenance procedures. In this study the maintenance recommendations of the manufacturer were followed: a monthly catheter flush with 5 mL of heparinized (100 IU/mL) normal saline. Instead, in the study without reported thrombosis [17] the system was flushed weekly with normal saline (3 mL) followed by a heparin lock of 1 mL of heparinized (1000 IU/mL) saline. More research is needed to specify whether more frequent flushing and/or increased heparin concentrations are most effective in decreasing catheter-associated thrombosis in canine VAP applications.

In our study, the presence of thrombi in the main stem of the portal vein of two dogs urged the premature removal of the VAP system. In human medicine, catheter removal because of thrombus formation is only considered if anticoagulation therapy fails or is contraindicated [22–26]. Because of missing evidence supporting successful use of thrombolytics in canine venous thrombosis and potential risk of inducing bleeding tendencies [27–30], it was decided to remove the VAP systems. Thrombectomy during celiotomy for removal of the VAP system was considered too risky [27]. After removal of the VAP system the dogs had an uneventful recovery. Further research is necessary to determine the optimal maintenance regime to prevent catheter-associated thrombosis in VAP systems in the canine portal vein.

Although the number of dogs in our study was limited, the results of this study could benefit other veterinary clinicians and researchers.

## Conclusions

This pilot study demonstrates that a VAP system can be effectively applied in dogs to obtain long term access to the portal venous system for venous pressure measurements, repeated infusions, and blood sampling. However, the long term presence of the device may increase the risk for dislodgement or thrombosis complications of the port.

## Data Availability

All data generated or analysed during this study are included in this published article.

## Abbreviations

aPTT: Activated partial thromboplastin time
PT: Prothrombin time
VAP: Vascular access port
